# Effectiveness of a cardiac rehabilitation program on biomechanical, imaging, and physiological biomarkers in elderly patients with heart failure with preserved ejection fraction (HFpEF): FUNNEL + study protocol

**DOI:** 10.1186/s12872-023-03555-7

**Published:** 2023-11-10

**Authors:** Antonio Ignacio Cuesta-Vargas, Iván José Fuentes-Abolafio, Celia García-Conejo, Estíbaliz Díaz-Balboa, Manuel Trinidad-Fernández, Daniel Gutiérrez-Sánchez, Adrián Escriche-Escuder, Lidia Cobos-Palacios, Almudena López-Sampalo, Jose Maria Pérez-Ruíz, Cristina Roldán-Jiménez, Miguel Angel Pérez-Velasco, Javier Mora-Robles, Mª Dolores López-Carmona, David Pérez-Cruzado, Jaime Martín-Martín, Luis Miguel Pérez-Belmonte

**Affiliations:** 1 Grupo de Investigación Clinimetría F14, Instituto de Investigación Biomédica de Málaga y Plataforma en Nanomedicina (IBIMA-Bionand)), IBIMA Plataforma-Bionand, Málaga, 29590 Spain; 2 Departamento de Fisioterapia, Facultad de Ciencias de La Salud, Universidad de Málaga, Andalucía Tech, Málaga, 29071 Spain; 3 Universidade da Coruña, Departamento de Medicina y Ciencias Biomédicas, Facultad de Fisioterapia, Campus de Oza, 15071 A Coruña, Spain; 4 Grupo de Cardiología, Instituto de Investigación Sanitaria de Santiago de Compostela (IDIS), 15706 A Coruña, Santiago de Compostela Spain; 5 Departamento de Enfermería, Facultad de Ciencias de La Salud, Universidad de Málaga, 29071 Andalucía TechMálaga, Spain; 6 Servicio de Medicina Interna, Hospital Regional Universitario de Málaga, Instituto de Investigación Biomédica de Málaga (IBIMA), Málaga, Spain; 7 Servicio de Cardiologia, Hospital Regional Universitario de Málaga, Instituto de Investigación Biomédica de Málaga (IBIMA), Málaga, Spain; 8 Area de Medicina Legal, Departamento de Anatomia Humana, Facultad de Medicina, Universidad de Málaga, Andalucía Tech, 29071 Málaga, Spain; 9 Centro de Investigación Biomédica en Red Enfermedades Cardiovasculares (CIBERCV), Instituto de Salud Carlos III, Madrid, Spain

**Keywords:** Heart failure, Preserved ejection fraction, Sarcopenia, Frail elderly syndrome, Cardiovascular rehabilitation, Functional physical performance

## Abstract

**Background:**

Patients with heart failure with preserved ejection fraction (HFpEF) have a low functional status, which in turn is a risk factor for hospital admission and an important predictor of survival in HFpEF. HFpFE is a heterogeneous syndrome and recent studies have suggested an important role for careful, pathophysiological-based phenotyping to improve patient characterization. Cardiac rehabilitation has proven to be a useful tool in the framework of secondary prevention in patients with HFpEF. Facilitating decision-making and implementing cardiac rehabilitation programs is a challenge in public health systems for HFpEF management. The FUNNEL + study proposes to evaluate the efficacy of an exercise and education-based cardiac rehabilitation program on biomechanical, physiological, and imaging biomarkers in patients with HFpEF.

**Methods:**

A randomised crossover clinical trial is presented among people older than 70 years with a diagnosis of HFpEF. The experimental group will receive a cardiac rehabilitation intervention for 12 weeks. Participants in the control group will receive one educational session per week for 12 weeks on HFpEF complications, functional decline, and healthy lifestyle habits. VO_2_peak is the primary outcome. Biomechanical, imaging and physiological biomarkers will be assessed as secondary outcomes. Outcomes will be assessed at baseline, 12 weeks, and 24 weeks.

**Discussion:**

Identifying objective functional parameters indicative of HFpEF and the subsequent development of functional level stratification based on functional impairment ("biomechanical phenotypes") may help clinicians identify cardiac rehabilitation responders and non-responders and make future clinical decisions. In this way, future pharmacological and non-pharmacological interventions, such as exercise, could be improved and tailored to improve quality of life and prognosis and reducing patients' hospital readmissions, thereby reducing healthcare costs.

**Trial registration:**

NCT05393362 (Clinicaltrials.gov).

**Supplementary Information:**

The online version contains supplementary material available at 10.1186/s12872-023-03555-7.

## Background

Cardiovascular diseases continued to be the principal cause of disability-adjusted life years (DALYs) due to non-communicable diseases (NCDs) and the leading cause of death, especially in countries with a higher sociodemographic index and longer life expectancy [1–3]. Within cardiovascular diseases, it has been estimated that heart failure (HF) has a prevalence of approximately 2% to 3%, arising in more than 23 million people worldwide [4, 5]. The incidence and prevalence of HF are increasing due to the ageing of the world population, with patients over 75 years of age having the highest risk of developing this condition [4, 6–8]. Within the complex entity of HF, three subtypes have been differentiated: HF with preserved ejection fraction (HFpEF), HF with mildly reduced ejection fraction (HFmrEF), and HF with reduced ejection fraction (HFrEF). These three diagnostic entities coexist in the population with HF, the most prevalent being HFpEF, with prevalence rates of 50%. HF constitutes the essential hospital diagnosis in older adults, being the leading cause of hospital admissions for people over 65 and contributing to the increase in healthcare costs in Western societies [4–6]. Functional status is a potentially modifiable risk factor for hospital admission and appears to be an important discriminator between the different HF subtypes [9].

HFpEF patients present distinctive functional characteristics, such as reduced aerobic capacity, decreased muscle strength in the lower extremities, low weekly physical activity, and exercise intolerance, accompanied by fatigue and dyspnoea symptoms [10–15]. These functional characteristics cause HF patients to show impaired functional abilities, experience impaired ability to perform activities of daily living, and suffer reduced quality of life [10, 12, 15]. Furthermore, it has been shown that patients with chronic HF present some changes in their gait pattern, with a lower gait speed than healthy subjects of the same age [16]. While gait speed is independently associated with survival, death, and hospitalisation in HF patients [8, 9, 17], maximal aerobic capacity has been inversely correlated with the severity of HF and directly related to prognosis and survival life expectancy [11, 12, 18, 19]. Similarly, lower extremity skeletal muscle mass and strength could predict long-term survival in patients with HF [12, 20].

When evaluating functional parameters in patients with HF, maximal oxygen consumption (VO_2_max) obtained from a cardiopulmonary exercise test (CPET) is considered the Gold Standard measure of cardiovascular functional capacity. Some functional tests have been used in clinical practice, such as the 6-min walk test (6MWT), which indirectly measures cardiovascular functional ability. Strength could also be assessed using the one repetition maximum (1RM) test, handgrip strength measurement (HGS), or the Short Physical Performance Battery (SPPB), as a valuable and indirect measure of this capacity [11]. Most geriatric populations present a high degree of fragility and dependence from the physical, cognitive, and psychological points of view, so their evaluation is essential [21]. SPPB and the Timed Up and Go (TUG) test have been established as practical clinical tests to assess frailty as a phenotype in HF patients [22–25].

By assessing the functional parameters used in the clinic, it has been possible to evaluate the effectiveness of Cardiac Rehabilitation (CR) in improving quality of life, functional capacity, exercise performance, and HF-related hospitalisations [26, 27]. CR is a multidimensional treatment designed to promote lifestyle changes and physical activity, optimise medical treatment, control risk factors, and address social and psychological problems following the development of heart disease [28]. CR programmes have a strong recommendation (Class 1A) in major HF practice guidelines, as it is considered a cost-effective intervention in HF, reducing recurrent hospitalisations and healthcare expenditure [7, 26, 29]. Benefits have also been shown on anthropometric, blood markers and physiological (VO_2_max) related outcomes, and cardiac imaging structural biomarkers such as ventricular ejection fraction [29–31].

Biomechanical biomarker assessment is possible through the parameterisation of human movement [32]. Inertial sensors have proven to be an accurate and reliable method for biomechanical human motion analysis and are used as a reference for validating motion capture instruments [32–35]. Currently, a good correlation between measurements obtained by inertial sensors and depth chambers has been demonstrated in the parameterisation of functional tests [34]. Biomechanical biomarkers could identify normal and pathological movements, the degree of impairment, the planning of rehabilitation strategies, and the evaluation of the effect of various interventions [32]. Therefore, it could be interesting to identify objective functional parameters affected in patients with HF, help stratify them based on different levels of functional impairment and identify those patients who are responders and non-responders to CR programmes. However, no biomechanical biomarkers have been assessed as an objective measure of the functionality of HF patients. Thus, the benefits of CR programmes on these biomechanical biomarkers have not been determined.

Facilitating decision-making and implementing CR programmes is a challenge in public health systems for HF management. This study proposes evaluating the efficacy of the CR programmes through a randomised clinical trial (RCT) on biomechanical, physiological, and imaging biomarkers in HF patients. In addition, it aims to validate the biomechanical analysis obtained using motion capture systems to assess objective functionality in elderly patients with HFpEF.

## Methods

### Trial design

**Fig. 1:**
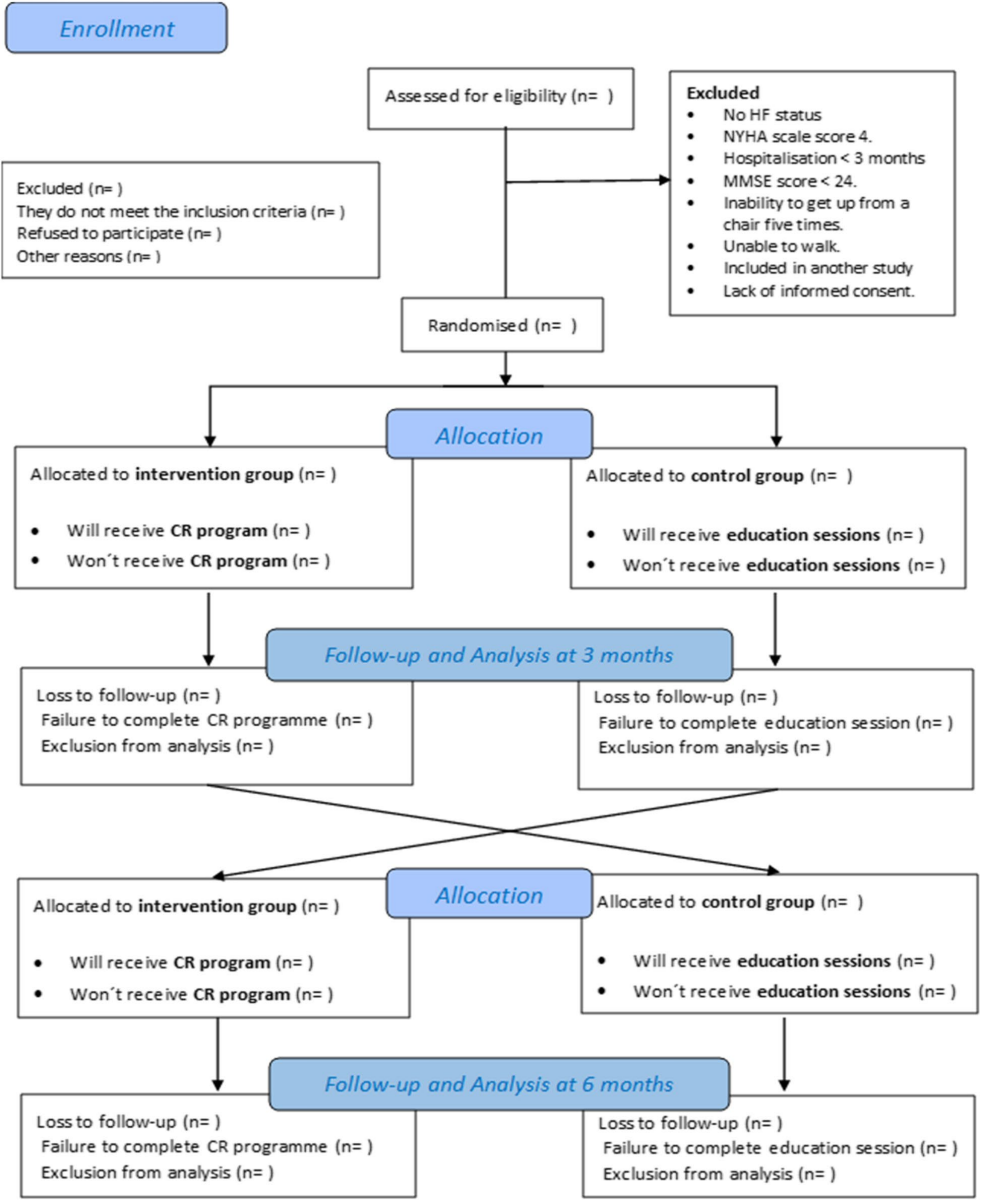
CONSORT Flow diagram; CR Cardiac Rehabilitation, HF Heart Failure, MMCSE Mini-Mental Cognitive Examination, NYHA New York Heart Association Classification

This study protocol followed the SPIRIT 2013 Recommendations [36] during its development. More details are shown in Additional file 1. The present study shows the proposed two-arm crossover RCT comparing an exercise-based CR programme with education in patients with HF. Patients will be randomised into two groups by simple randomisation using a random number generator on the computer. The study design is shown in Fig. 1. The RCT is based on work within the Biomedical Research Institute of Malaga (IBIMA) between the Internal Medicine Unit and the Cardiology Unit of the Regional University Hospital of Malaga (HRUM), together with the support of the University of Malaga (Malaga, Spain). This project has been submitted to the Malaga Provincial Ethics Committee (2198-N-22).

### Study duration and timing

**Fig. 2:**
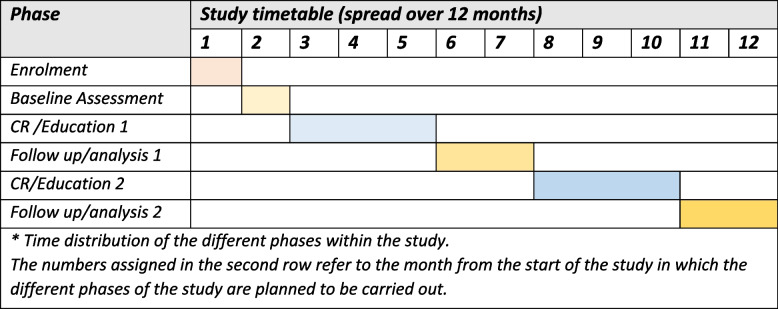
Study timetable; * Time distribution of the different phases within the study. The numbers assigned in the second row refer to the month from the start of the study in which the different phases of the study are planned to be carried out

Recruitment and eligibility assessment of participants from the Internal-Cardiac Unit will be performed first. Patients who meet the eligibility criteria and wish to participate in the study will be required to provide written informed consent to participate in the study. Once the informed consent is signed, study variables will be collected. After baseline assessment, patients will be randomised to the control (Education) or intervention (CR) group. The interventions will have an estimated duration of 3 months. Patients will be assessed right after the intervention (follow-up/analysis 1) and at six months (follow-up/analysis 2). This way, depending on the group allocation, the different intervention groups will be reassigned. Data collection is expected to be completed about 1 year after the beginning of the study. The timeline of the study is represented in Fig. 2.

### Recruitment and inclusion

They will be patients over 70 years of age with a diagnosis of HFpEF, who are clinically stable, and who are being followed in this unit. A clinically stable patient is defined as a patient who has not been hospitalised for HF decompensation, has not undergone treatment modification, and has a stable NYHA grade 2–3 in the last 3 months. The diagnosis of HF and optimal treatment will be established according to Spanish and European HF clinical practice guidelines [29, 37]. Inclusion and exclusion criteria are detailed in Table 1.

**Table 1:** Inclusion and exclusion criteria

*Inclusion criteria*	*Exclusion criteria*
Age 70 years or older	Cardiac pathologies that do not have HF status
Diagnosis of HFpEF followed in the unit Intention to change sedentary behaviours expressed by the subject	NYHA scale score 4
Clinical & haemodynamic stability	Have been hospitalised before three months or less
To Receive optimal medical treatment	MMSE score less than 24
	Inability to get up from a chair five times
	Unable to walk or independently without a walking aid (cane, crutch or walker)
	To participate in an experimental study where they receive treatment
	Lack of informed consent

*HF* Heart Failure, *MMSE* Mini-Mental State Examination, *NYHA* New York Heart Association

## Interventions

Both the Education and CR interventions will take place in the internal medicine unit of the HRUM. Medical and nursing care will be available during Education and CR sessions to ensure the safety of participants Study collaborators will be instructed to record any incidents related to possible adverse events that may be associated with the interventions.

### Experimental group: CR program

The CR programme will consist of aerobic and strength exercise sessions. Exercises will be individualised after assessment of short (strength exercise) and long (aerobic exercise) efforts. They will be performed two days a week and at least 48 h between sessions. Progression will consider a clinical criterion, determined by the absence of HF symptoms at the current intensity, and a time criterion in which, as long as the clinical criterion is met, the intensity will be increased every two to three weeks. The progression in aerobic training intensity is established based on Skinner's three-phase model [38] and the recommendations of current European cardiac rehabilitation guidelines [29]. The structure of the standard session, the criteria for progression in both aerobic and strength training, the phases of training, the periodisation of the programme, and the monitoring variables in the session can be found in detail in Additional file 2.

In addition, embedded in the exercise intervention, education on healthy lifestyle habits will be needed. A series of competencies in healthy lifestyle habits relevant to the population with HF will be established, which will be evaluated using a questionnaire with an evaluation rubric format for each of the competencies. The competencies will be evaluated during the training sessions in an informal way. Depending on the results obtained, they will be reinforced by strengthening unconsolidated skills using specific educational material.

### Control group: Education

The control group will receive personalized educational embedded in the program for twelve weeks on HF complications, functional decline, and healthy lifestyle habits. The format of the sessions will be in the form of face-to-face or online master classes, depending on the availability of the participants, in which the active participation of both volunteers and their relatives/caregivers will be encouraged.

### Criteria for dropout

The criteria for discontinuing the assigned interventions are:

1. The patient's willingness to discontinue participation in the study.
2. Worsening prognosis that precludes continuation.
3. The presence of events during the sessions jeopardizes the patient's safety.
4. Absence from training sessions, in the case of the experimental group for unjustified reasons (compliance below 60%).

### Study outcomes

Anthropometric and demographic variables will be collected for descriptive analysis only in the first evaluation, including age, gender, weight, height, and body mass index. Participants must complete different questionnaires that will be conducted at each measurement time. The summary of the outcome variables can be found in Additional file 3.

#### Primary outcome measures

As primary variables, one has been chosen for each of the three domains that are going to be evaluated.

##### Biomechanical biomarkers

The kinematic parameters during the activity are derived by using inertial measurement units (IMU). IMUs have been shown to have good psychometric properties to estimate kinematic parameters during the performance of functional tests. Kinematic parameters have been seen as objective parameters of functional capacity. It has been shown to have high specificity and sensitivity for tests such as the 6MWT in patients with HF.

##### Physiological biomarkers

Peak oxygen uptake (VO_2_peak), in maximal exercise tests, coincides with maximal oxygen consumption (VO_2_max). It is the most objective parameter of functional capacity and a gold standard indicator of maximal cardiorespiratory fitness. It is the maximum O_2_ extracted from inhaled air during pulmonary ventilation. It is usually expressed in milliliters per minute (ml/min) or milliliters per kilogram per minute(ml/kg/min). It reflects the severity of the disease in patients with HF [39, 40] and is a significant predictor of mortality in HF patients [41].

##### Imaging biomarkers

Assessment of ultrasonic body composition parameters can be performed using ultrasonography (US). This technique is reliable and valid for evaluating the number of pennate muscles in older adults, such as the quadriceps femoris (QF) muscle. US has shown reasonable validity in estimating muscle mass compared to MRI and CT. US could have the potential for use in clinical practice for the detection of sarcopenia and to assess body composition or muscle architecture.

#### Secondary outcome measures

For the secondary variables, we have chosen to describe the instruments used to capture the different variables. The variables assigned to each instrument are well-detailed in Additional file 3.

### Objective outcome variables

#### Physiological biomarkers

##### Electrical impedance vector analysis (BIVA)

As a measure of body composition, electrical bioimpedance vector analysis (BIVA) has been validated. It has recently been shown to provide information on the overall assessment, management, and prognostic evaluation of HF patients [42, 43]. All measurements derived from BIVA are characterised by excellent test–retest reliability [44]. The BIO 101 BIVA®PRO bioimpedance device (BIA101 Akern, 50,065 Pontassieve FI, Italy) will be used.

##### Respiratory function tests (RFT)

To measure the patient's respiratory volumes and flows, a spirometry device (Spiro USB, Micro Medical, Kent, UK) [45] and a respiratory pressure meter (MicroRPM, Micromedical, Kent, UK) [46] will be used to test the lung function [46–48]. The repeatability of spirometry measurements [49] and the reliability of the MicroRPM device for MIP and MEP [50] variables have been reported. The recommendations of the ARTP statement on pulmonary function tests in 2020 will be followed [51].

##### High-density electromyography (HD-sEMG)

The high-density electromyography (HD-sEMG) device will be used in conjunction with the S-type load cell (Biometrics Ltd., Newport, UK) to measure the electrical activity of the tibialis anterior (TA) muscle and generate force output. It is a reliable method for assessing the characteristics of the motor unit in different populations and locations [52–55]. The signal produced will be recorded using the external analog-to-digital signal converter Sessantaquattro (64-channel EMG amplifier; OT Bioelettronica, Turin, Italy), and will be processed using OT Biolab + software (v.1.2.1, OT Bioelettronica, Turin, Italy) [56].

##### Functional and dynamometric tests (FUN test)

Patients will undergo the Short Physical Performance Battery (SPPB) [57–59], Timed Up and Go Test (TUG) [57, 60], and Six-Minute Walk Test (6-MWT) [58, 59, 61, 62]. A digital hand dynamometer will assess isometric quadriceps extension strength (J Tech Medical, Powertrack II Commander, Salt Lake City, UT) [63]. Grip strength will be evaluated using the Jamar Hydraulic, model SH5001 (Lafayette Instrument, Lafayette, USA). Manual dynamometry has proven to be a valid and reliable instrument to measure isometric strength in different populations and locations [64, 65].

##### Cardiopulmonary Exercise Tests (CPET)

Cardiopulmonary exercise testing (CET) will be performed using a portable Cortex Metamax 3B (MM3B) [66] automated gas analysis system[ref]. Primarily to obtain VO_2_peak, secondary to collect variables derived from breath-by-breath respiratory gas analysis. The self-limited ramped exercise protocol on an ergometer bike is generally well tolerated by patients with HF [67]. The feasibility and safety of the ramped exercise protocol have been demonstrated [68].

##### Blood biomarkers

The relevant physician will extract biomarkers in the blood through blood analysis (blood, plasma, and serum). The central blood values of interest in the HF population will be requested.

#### Biomechanical biomarkers

During functional tests, biomechanical parameters will be assessed using the inertial measurement unit of the Shimmer3 [69] and the structured light-based depth camera manufactured by Xbox360, the Kinect 2.0 sensor [35, 70, 71].

#### Imaging biomarkers

Standard two-dimensional resting echocardiography (ultrasound) will be used to obtain cardiac imaging variables. Simpson's biplane method will be used in an apical four-chamber view to estimate LVEF (%) [29, 31]. A B-mode ultrasound (SonoSite 180 Plus, SonoSite Japan, Tokyo, Japan) and linear transducer (5–10 MHz) [72] will be used to collect skeletal and pulmonary imaging variables.

### Self-reported outcome variables

The abbreviated comprehensive assessment scale (aCGA) [73, 74], the SARC-F questionnaire [75, 76], the Kansas City questionnaire (KCCQ) [77], and the Mini Nutritional Assessment survey (MNA®) [78] are validated and, will be used to obtain the outcome variables self-reported.

### Exploratory outcomes

During the sessions, the total exercise time and the exercise intensity reached by the patients during the sessions will be collected. In addition, adverse events during the exercise program will be compiled.

The cost of the CR per session will be estimated from the material required, the clinician's time, and the indirect costs.

### Data management

The data will be recorded and stored in database files on a password-protected flash drive. No identifying information is registered in the database, but an identification number is attributed to each patient. This number is associated with the patient's medical record number in a separate table accessible only to FUNNEL + researchers on a corporate computer. All patient information will be pseudonymised and only researchers will have access to the identification data.

### Blinding

The researchers conducting the assessments and data analysis will be blinded so that they do not know to which group each patient belongs. Blinding the subjects is unfeasible because of this type of intervention.

### Sample size calculation

The sample size was calculated using G Power 3.1.9.2 software (University of Düsseldorf, Germany). Based on an expected difference in VO_2_max increase of 3.0/ml/kg/min in the intervention group and 0.6 ml/kg/min in the control group (based on the result of the EUCaRE Study, Prescott et al. 2020 PMID 32102550 [48]). Assuming a statistical power of 80% and an alpha error of 0.05, the required sample size is 55 patients per arm and carries a loss rate of 15%, and this would make a total of 126 (63 per arm).

### Statistical analysis

Data sets will be processed using the Statistical Package for the Social Sciences (SPSS) for Windows (version 19.0, SPSS Inc., Chicago, IL, USA). Qualitative variables will be described by absolute and relative frequency (percentage). Quantitative variables shall be presented by the mean and standard deviation if the data follow a normal distribution or by the maximum, minimum, and three quartiles if the data do not follow such a distribution. The Shapiro–Wilk test will be used to analyse the normal distribution of the data (*p* > 0.05). The difference in means between the two groups will be obtained using Pearson's Chi-square test (× 2) for qualitative variables. To express the differences in means in the quantitative variables between the two groups, the bivariate analysis will be used using the parametric Student's t-test for independent samples in the case of normal data distribution, or the non-parametric Mann–Whitney U test, if there is no normal distribution of the data. The degree of statistical significance shall be set at a *p*-value < 0.05. 95% confidence intervals (CI) will be calculated for the mean differences between the study groups.

### Dissemination

The trial findings will be submitted for publication in an unrestricted peer-reviewed clinical journal and reported at pertinent conferences.

## Discussion

The aim of this study is to evaluate the efficacy of CR programs through a randomized clinical trial (RCT) on biomechanical, physiological and imaging biomarkers in patients with HFpEF.

Previous cross-sectional studies [79–81] have assessed objective functional parameters, providing interesting results that have allowed the estimation of VO_2_peak and objective measurement of dyspnoea and fatigue in patients with HFpEF.

Although the term HFpEF is used to describe a category of HF, there is great variability in the clinical presentation and progression of the disease in individual patients. For example, patients with HFpEF may present with symptoms such as dyspnoea, fatigue, peripheral oedema, and a wide variety of comorbidities, such as chronic kidney disease, hypertension, diabetes mellitus, and obesity, which can affect disease progression and response to treatment. Patients may require additional treatments to manage these conditions depending on the comorbidities present. Therefore, phenotyping of patients with HFpEF is essential to determine the best treatment strategy [82, 83].

Phenotyping refers to identifying subgroups of patients with common clinical and biological characteristics. In the case of HFpEF, phenotyping may help identify subgroups of patients with different causes, comorbidities, risk factors, and disease severity. Current literature describes the association of HFpEFF with diabetes mellitus, obesity, pulmonary disease, or frailty and proposes treatment strategies according to the different presentations [82, 83].

**Fig. 3:**
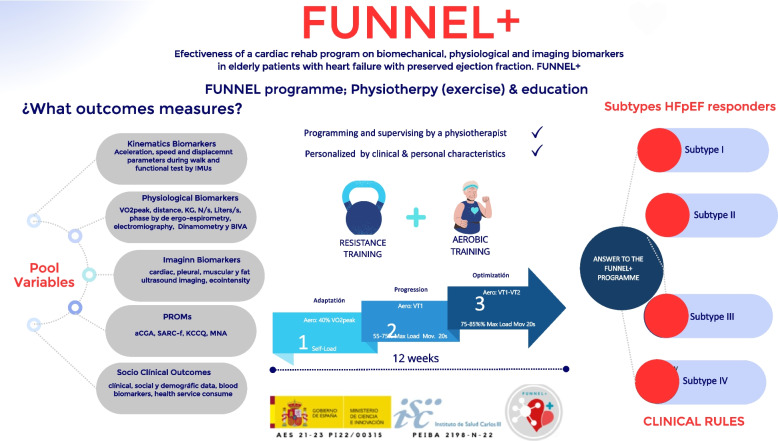
Subtypes responder to the Funnel program

However, although the poor functional capacity of patients with HF, especially in HFpEF, is well known, no phenotypes describing the functional status of these patients for subsequent treatment prescription have been reported so far. Through the present study, we aim to identify objective functional parameters indicative of HF. The subsequent development of a functional level-based classification ("biomechanical phenotypes") may help clinicians identify CR responders and non-responders and thus make future clinical decisions (Fig. 3. Subtypes responder to the Funnel program). In this way, future pharmacological and non-pharmacological interventions, such as exercise, could be improved and tailored, improving quality of life and prognosis and reducing patients' hospital readmissions, thereby reducing healthcare costs.

### Supplementary Information

**Additional file 1.** SPIRIT_Fillable-checklist.

**Additional file 2.** Summary of CR programme.

**Additional file 3.** Summary of Study outcomes.

## Data Availability

Data are accessible under justifiable request at https://riuma.uma.es/xmlui/.

## Abbreviations

aCGA: Abbreviated Comprehensive Assessment Scale
ARTP: Association for Respiratory Technology and Physiology
BIVA: Electrical Bioimpedance Vector Analysis
CPET: Cardiopulmonary Exercise Test
CR: Cardiac Rehabilitation
HD-sEMG: High-Density Electromyography
HGS: Hand Grip Strength
HF: Heart Failure
HFpEF: Heart Failure With Preserved Ejection Fraction
KCCQ: Kansas City Questionnaire
LVEF: Left Ventricular Ejection Fraction
MEP: Maximum Expiratory Pressure
MIP: Maximum Inspiratory Pressure
MMSA: Mini-Mental State Examination
MNA®: Mini Nutritional Assessment survey
NCDs: Non-Communicable Diseases
NYHA: New York Heart Association
SARC-F: SARC-F questionnaire
SPPB: Short Physical Performance Battery
SPSS: Statistical Package for the Social Sciences
TUG: Timed Up and Go
VO_2_max: Maximal Oxygen Consumption
VO_2_peak: Peak Oxygen Uptake
1-RM: One-Repetition Maximum
6-MWT: Six-Minute Walk Test
