# Supplementary material for: Effectiveness of a cardiac rehabilitation program on biomechanical, imaging, and physiological biomarkers in elderly patients with heart failure with preserved ejection fraction (HFpEF): FUNNEL + study protocol
Source: BMC Cardiovasc Disord. 2023 Nov 10;23:550. doi: 10.1186/s12872-023-03555-7 (PMC10638727; doi:10.1186/s12872-023-03555-7)
Supplement: Supplementary file 2 — Additional file 2. Summary of CR programme. [file 12872_2023_3555_MOESM2_ESM.docx]

**Additional file 2 .Summary of CR programme**

1. **Main components of the exercise program**

The main components of the exercise program, as well as the structure of each one of the exercise sessions, are specified in Table 1 and Table 2, respectively**.**

Table 1. Main component of exercise program

| **Number of session** | 24 sessions scheduled in 12 weeks |
| --- | --- |
| **Frequency** | 2 days per week |
| **Intensity** | *Aerobic exercise: 40-85%* of maximum oxygen consumption *(*VO_2_peak)  *Strength exercise: 55-85%* of maximum load mobilised in 20 seconds (MLM-20s) |
| **Time** | 60 min/day |
| **Type** | Aerobic, strength and respiratory exercise |

Table 2. Structure of exercise session

| **Structure of session** | | | | **Time spent** | |
| --- | --- | --- | --- | --- | --- |
| **1** | *Warm up* | Breathing and joint mobility exercises seated and standing | | 5-10 min | |
| **2** | *Main part* | 2.1 | Strength circuit training based on functional movements (e.g. sit-stand, pull, push) with a progressive increase in workload using bodyweight, dumbbells, kettlebells and elastic bands. | 14 min – 30 min | 40-45 min |
|  |  | 2.2 | Aerobic training on a treadmill or recumbent bike. SIT and LIT will alternate weekly. | SIT:13 min -18 min  LIT: 20 min-28 min |  |
| **3** | *Cool down* | Return to calm seated: gentle stretching and calming breathing | | 1-5 min | |
| ***Total*** | | | | **60 min** | |
| ***Abbreviation****: Short-Interval training* (SIT) and *Long-Interval training* (LIT) | | | | | |

1. **Progression criteria**

Training progression will be individualised within each session, according to the patient's physical condition and symptoms (dyspnoea, fatigue, pain) during exercise. The criteria for progression will depend on compliance with the prescribed time (total session duration) and intensity of exercise (based on VO2peak and MLM-20s), as described in Table 3.

Table 3. Criteria for progression in based time and intensity of exercise

|  | *Strength training* | | *Aerobic training* | |
| --- | --- | --- | --- | --- |
| Time | Sets and movements | 4 sets, 2-4 movements, combining upper and lower limb exercises | Sets and work time | SIT: 10-12 sets of 20-30 sec  LIT: 3-4 sets of 4 min |
|  | Work and rest time | 20 sec work, 90 sec rest | Exercise density | 1:2/1:1/2:1 |
| Intensity | 1st Month | 65% of MLM-20s | 1st Month | 65% of VO_2_peak |
|  | 2nd Month | 75% of MLM-20s | 2nd Month | 75% of VO_2_peak |
|  | 3rd Month | 85% of MLM-20s | 3rd Month | 85% of VO_2_peak |
| **Note:** The progression of the exercise will be first in time and then in intensity.  **Abreviations**: Short-Interval training(SIT);Long-Interval training (LIT); Aerobic training (AT); Strength training (ST); TL(Training Load);Peak oxygen consumption(VO_2_peak);Maximum load mobilised for 20 seconds(MLM-20s); movements (mvs); active recovey (ar); s(seconds);min (minutes) | | | | |

1. **Periodization of the exercise program**

The periodisation of exercise in the cardiac rehabilitation program is shown in Table 4. Three mesocycles have been established to differentiate the following phases:

- *Adaptation phase:*
- Workload under VT1 threshold *(40% of VO2peak, RPE 11-12).*
- Duration: 2 weeks (from 1 to 2 week)
- *Progression phase:*
- Workload at VT1 threshold *(50%-65% VO2peak; RPE 12-13)*
- Duration: 4 weeks (from 3 to 7 week)
- *Optimisation phase:*
- Workload between VT1 and VT2 thresholds *(65%-85% VO2peak; RPE 14-16)*
- Duration: 6 weeks (from 8 to 12 week)

Table 4. Periodization of the exercise program

|  |  |  | *Strength training* | *Aerobic training* | |
| --- | --- | --- | --- | --- | --- |
| *Month* | *Phase* | Week | Strenght training  Session 1 and 2 | Aerobic training  Session 1 (SIT) | Aerobic training  Session 2 (LIT) |
| 1st Month | Adaptation | 1 | 2-4 mvs, 4 sets of 20s work/90s rest, with bodyweights | 10- 12 sets o 20-30s work,40% of VO_2_peak and 40-60 seg of a.r  RPE work 11–12 | 3-4 sets of LIT (4min of work,TL 40% of VO_2_peak)  RPE work 11–12 |
|  |  | 2 | 2-4 mvs, 4 sets of 20s work/90s rest, with bodyweights | 10- 12 sets of 20-30s of work, 40%-50% of VO_2_peak and 40-60s of a.  RPE 12-13 | 3-4 sets of 4min of work,TL 40%-50% of VO_2_peak  RPE work 11–12 |
|  | Progression | 3 | 2-4 mvs, 4 sets of 20s work/90s rest, 55%- 65% of MLTM-20s | 10- 12 sets of 20-30s of work, 50%-60% of VO_2_peak and 40-60s of a.r  RPE 12-13 | 3-4 sets of 4min of work, 50%-60% of VO_2_peak)  RPE 12-13 |
|  |  | 4 | 2-4 mvs, 4 sets of 20s work/90s rest 55%- 65% of MLTM-20s | 10- 12 sets of 20-30s of work, 50%-60% of VO_2_peak and 40-60s of a.r  RPE 12-13 | 3-4 sets of 4min of work, 50%-60% of VO_2_peak)  RPE 12-13 |
| 2nd Month |  | 5 | 2-4 mvs, 4 sets of 20s work/90s rest 55%- 65% of MLM-20s | 10- 12 sets of 20-30s of work, 55%-65% of VO_2_peak and 40-60s of a.r)  RPE 12-13 | 3-4 sets of 4min of work,TL 55%-65 % of VO_2_peak)  RPE 12-13 |
|  |  | 6 | 2-4 mvs, 4 sets of 20s work/90s rest 55%- 65% of MLM-20s | 10- 12 sets of SIT (20-30s of work,55%-65% of VO_2_peak and 40-60s of a.r)  RPE 12-13 | 3-4 sets of 4min of work,55%-65% of VO_2_peak  RPE 12-13 |
|  | Optimisation | 7 | 2-4 mvs, 4 sets of 20s work/90s rest 65%- 75% of MLM-20s | 10- 12 sets of SIT (20-30s of work,65%-75 % of VO_2_peak and 40-60sof a.r)  RPE 14-16 | 4-6 s of 4min of work, 65%-75% of VO_2_peak  RPE 14-16 |
|  |  | 8 | 2-4 mvs, 4 sets of 20s work/90s rest 65%- 75% of MLM-20s | 10- 12 sets of SIT (20-30s of work,65%-75 % of VO_2_peak and 40-60sof a.r)  RPE 14-16 | 3-4 sets of LIT (4min of work, 65%-75% of VO_2_peak  RPE 14-16 |
| 3rd Month |  | 9 | 2-4 mvs, 4 sets of 20s work/90s rest , 65%- 75% of MLM-20s | 10- 12 sets of SIT (20-30s of work, 70%-80% of VO_2_peak and 40-60s of a.r < 20 W) RPE 14-16 | 3-4 sets of 4min of work,TL 70%-80% of VO_2_peak  RPE 14-16 |
|  |  | 10 | 2-4 mvs, 4 sets of 20s work/90s rest ,65%- 75% of MLM-20s | 10- 12 sets of SIT (20-30s of work,70%-80% of VO_2_peak and 40-60s of a.r < 20 W) RPE 14-16 | 3-4 sets of 4min of work,TL 70%-80% of VO_2_peak  RPE 14-16 |
|  |  | 11 | 2-4 mvs, 4 sets of 20s work/90s rest , 75%- 85% of MLM-20s | 10- 12 sets of SIT (20-30s of work, 75%-85% of VO_2_peak and 40-60s of a.r < 20 W) RPE 14-16 | 3-4 sets of (4min of work,TL 75%-85% of VO2peak  RPE 14-16 |
|  |  | 12 | 2-4 mvs, 4 sets of 20s work/90s rest,75%- 85% of MLM-20s | 10- 12 sets of SIT (20-30s of work,75%-85% of VO_2_peak and 40-60s of a.r < 20 W) RPE 14-16 | 3-4 sets of 4min of work,75%-85% of VO_2_peak  RPE 14-16 |
| **Abreviations**: Short-Interval training(SIT);Long-Interval training (LIT); Aerobic training (AT); Strength training (ST); TL(Training Load);Peak oxygen consumption(VO2peak);Maximum load mobilised for 20 seconds(MLM-20s); movements (mvs); active recovey (ar); s(seconds);min (minutes) | | | | | |

1. **Monitoring**

The parameters that will be monitored before, during and post-exercise are detailed in Table 5.

Table 5. Monitoring parameters of exercise

| **Monitoring Parameters** | | **Timming** | | |
| --- | --- | --- | --- | --- |
|  |  | Pre-x | On-x | Post-x |
| *Objective monitoring parameters* | Heart Rate (HR) |  |  |  |
|  | Blood Pressure (BP) |  |  |  |
|  | Lactate levels |  |  |  |
|  | Blood glucose level |  |  |  |
| *Subjective monitoring parameters* | Rate of Perceived exertion (RPE) |  |  |  |
|  | Talk test |  |  |  |
|  | OMNI-Resistance Exercise Scale (OMNI-RES) of perceived exertion |  |  |  |
| ***Abbreviation****: Before exercise (pre-x); During exercise (on-x); After exercise (post-x)* | | | | |
