# Supplementary material for: Effectiveness of a cardiac rehabilitation program on biomechanical, imaging, and physiological biomarkers in elderly patients with heart failure with preserved ejection fraction (HFpEF): FUNNEL + study protocol
Source: BMC Cardiovasc Disord. 2023 Nov 10;23:550. doi: 10.1186/s12872-023-03555-7 (PMC10638727; doi:10.1186/s12872-023-03555-7)
Supplement: Supplementary file 3 — Additional file 3. Summary of Study outcomes. [file 12872_2023_3555_MOESM3_ESM.docx]

| *DOMAINS* | | *INSTRUMENTS* | *EXTRACTED VARIABLES [unit]* |
| --- | --- | --- | --- |
| Objective Variable | *Physiological biomarkers* | CPET | **VO_2_peak [ml/kg/min];** HRpeak[beats/min], Wpeak[Watts], CRE, VEpeak[breath/min]; ER, BR, VO2/HR, VE/VCO2, PETCO2, VO2/WR, VO2-VT1[ml/kg/min], OUES, Vd/Vt |
|  |  | Bioimpedance | Pha[º], R, Xc, FFM, TBW, ECW, BCM, FM, ECW/TBW |
|  |  | RFT | IC, FVC, VC, FEV_1_, IRV, ERV, MIP, MEP, VT |
|  |  | Electromyography | Adf, Ps |
|  |  | Dynamometry | Functional test: SPPB[points], TUG[s], 6MWT[m]  Digital hand dynamometer: Iso-S-QE-dyn[N]  Grip Dynamometer; Iso-S-HG[Kg]  Iso-S-DF[Kg], |
|  |  | Blood tests | Haemoglobin, hematocrit, MCV, MCH, MCHC, Leukocytes, Platelet count, Glucose, Creatinine, GFR, Uric Acid, Na, K,Ca, Tr-ALT,Tr-AST, Tr-GGT,Tr-FA, glycated hemoglobin, ferritin, iron, I-TFS, Transferrin, folic acid, TSH, TC, LDL, HDL, triglycerides, albumin, NT-proBNP, D,B_12_ |
|  | *Biomechanical biomarkers* | Shimmer[IMU] | **Step Time [s];** Gait Speed [m/s]; Steps [n]; Step Length [m]; Step Velocity [m/s]; Step Cadence [Steps/Min]; Step Symmetry Ratio [Step Time Max/Step Time Min]; Strides [n]; Stride Time [s]; Stride Length [m]; Stride Velocity [m/s]; Stride Cadence [Strides/Min]; Stride Symmetry Ratio [Stride Time Max/Stride Time Min] |
|  |  | Depth-camera [IMU] |  |
|  | *Imaging biomarkers* | Echo-cardiogram | LVEF[%], LV dilation [mm], LA diameter [mm], LA diameter [mm] |
|  |  | Echo-muscular | **FT-Q[cm],** MT-Q, FEI-Q, MEI-Q |
|  |  | Echo-Pulmonary | MT-DT-ins [cm], MT-DT-Esp [cm], Slide-Pl [cm] |
| Self-administers variables | | Questionnaires | aCGA [points], SARC-F [points], KCCQ. MNA®[points] |
| Exploratory variable | |  | Physical exercise time [min/ss]  Safety of intervention [number of advent events]  Unit Cost of CR [cost per ss] |
| Note [Primary outcome]: The highlighted variables in the table correspond to the primary variables within each of their domains.  Abbreviations:Extracellular water[ECW];Total body water[TBW];Phase angle[Pha];Left atrium[LA];Short physical performance battery [SPPB];Inspiratory Capacity [IC];Forced Vital Capacity [FVC];Slow Vital Capacity [VC]; Respiratory Coefficient or Respiratory Exchange Ratio [RER];Ventilatory Threshold Oxygen Consumption [VO2-VT1];Peak Oxygen Consumption [VO2 Peak];Kansas City Questionnaire [KCCQ];SARC-F Questionnair [SARC-F];Pleura-Lung Slide [Slide-Pl];DT [diaphragm]; Subcutaneous fat echo-intensity [FEI];Muscle echo-intensity [MEI];Aerobic efficiency [ΔVO2/ ΔWR];Mini Nutritional Assessment Survey [MNA®];Ventilatory carbon dioxide equivalent [VE/VCO2];Ventilatory oxygen equivalents [VE/VO2]; Abbreviated Comprehensive Geriatric Assessment Scale [aCGA]; Expiratory [Esp]; Peak Expiratory Flow [PEF]; Left Ventricular Ejection Fraction [LVEF]; Isometric strength hand grip [Iso-S-HG]; Isometric strength dorsiflexion with S-type gauge [Iso-S-DF]; Isometric strength quadriceps extension with manual dynamometer [Iso-S-QE-dyn];Subcutaneous fat thickness [FT];Muscle thickness [MT];Inspiration [ins];Body cell mass [BCM]; Fat mass [FM]; Fat free mass [FFM]; Oxygen Uptake Efficiency Slope [OUES]; Peak Expiratory Pressure [MEP]; Peak Inspiratory Pressure [MIP]; Partial Pressure of Carbon Dioxide [PETCO2]; Partial Pressures of Oxygen [PETO2]; Six Minute Walk Test [6-MWT]; Timed Up and Go Test [TUG]; Respiratory function tests [RFT]; Pulse Oxygen [VO2/HR]; Chronotropic response to exercise [CRE]; Reactance [Xc]; Quadriceps anterior rectus [Q]; Extracellular to total water ratio [ECW/TW]; Dead space to tidal volume ratio [Vd/Vt]; Respiratory reserve [BR] Resistance [R]; Peak work rate [Wpico]; Peak ventilation [VEpeak]; Left ventricle [LV]; Expiratory reserve volume [ERV]; Inspiratory reserve volume [IRV]; Forced expiratory volume in the first second [FEV1]; Tidal volume [VT];Respiratory function tests [RFT]; Average discharge frequency [Adf]: Propagation speed [Ps]; Mean Corpuscular Volume [MCV]; Mean Corpuscular Hemoglobin [MCH], Mean Corpuscular Hemoglobin Concentration [MCHC]; Glomerular Filtration Rate [GFR]; sodium[Na];potassium[K], calcium [Ca], transaminases [Tr]; Index of transferrin saturation [I-TFS]; vitamin B12[B12]; Total cholesterol [TC]; vitamin D[D]. | | | |

Additional file 3.Summary of Study outcomes
